# Intractable Vomiting in an 11-Month-Old Boy With Trisomy 21: A Case Report on Abnormal Calcium/Calcinosis/Creatinine in Down Syndrome

**DOI:** 10.7759/cureus.16827

**Published:** 2021-08-02

**Authors:** Minh Nguyen, Florentina Litra, Ammar Kamil, Berrin Ergun-Longmire

**Affiliations:** 1 Internal Medicine/Pediatrics, Western Michigan University Homer Stryker M.D. School of Medicine, Kalamazoo, USA; 2 Pediatrics, University of Florida, Pensacola, USA; 3 Pediatrics, Ascension Sacred Heart, Pensacola, USA; 4 Pediatrics, Sacred Heart Hospital, Pensacola, USA; 5 Pediatrics and Adolescent Medicine, Western Michigan University Homer Stryker M.D. School of Medicine, Kalamazoo, USA

**Keywords:** acute kidney injury, down syndrome, acute hypercalcemia, intractable vomiting, calcinosis

## Abstract

Abnormal calcium/calcinosis/creatinine in Down syndrome (ABCD syndrome) is a very rare condition with no clear etiology. In this paper, we describe our clinical encounter with this disease. We report the case of an 11-month-old male infant with Down syndrome (DS) who presented to the hospital with intractable vomiting and decreased oral intake and urine output. The evaluation revealed an acute kidney injury (AKI) and hypercalcemia. Although his AKI improved with intravenous hydration, his hypercalcemia persisted. Extensive studies were notable for an elevated urinary excretion of calcium and bilateral medullary nephrocalcinosis seen on renal ultrasound (US). As a result, he was diagnosed with ABCD syndrome. Dietary calcium restriction was implemented. During his follow-up visit with a pediatric endocrinologist, his serum calcium was found to be normalized. To our knowledge, this is only the seventh case report on ABCD syndrome in the literature.

## Introduction

Hypercalcemia is defined as a condition where serum calcium level is greater than two standard deviations above the mean [1]. Normal serum levels of calcium are achieved through a close interaction between the parathyroid, kidneys, and bones. This finding is observed less commonly in children than in adults. The differential diagnosis of hypercalcemia varies depending on the age group: in neonates and infants versus children over two years of age and adolescents. In the neonatal group, Down syndrome (DS) is considered to be an etiology for hypercalcemia [2]. Specifically, there is abnormal calcium/calcinosis/creatinine in Down syndrome or ABCD syndrome, which involves a triad of hypercalcemia, hypercalciuria, and nephrocalcinosis. Overall, this is a rare disease with only six case reports in the current literature. In this report, we present an 11-month-old DS patient who was diagnosed with ABCD syndrome. This study adds to the growing body of literature on this challenging diagnosis.

## Case presentation

**Figure 1:**
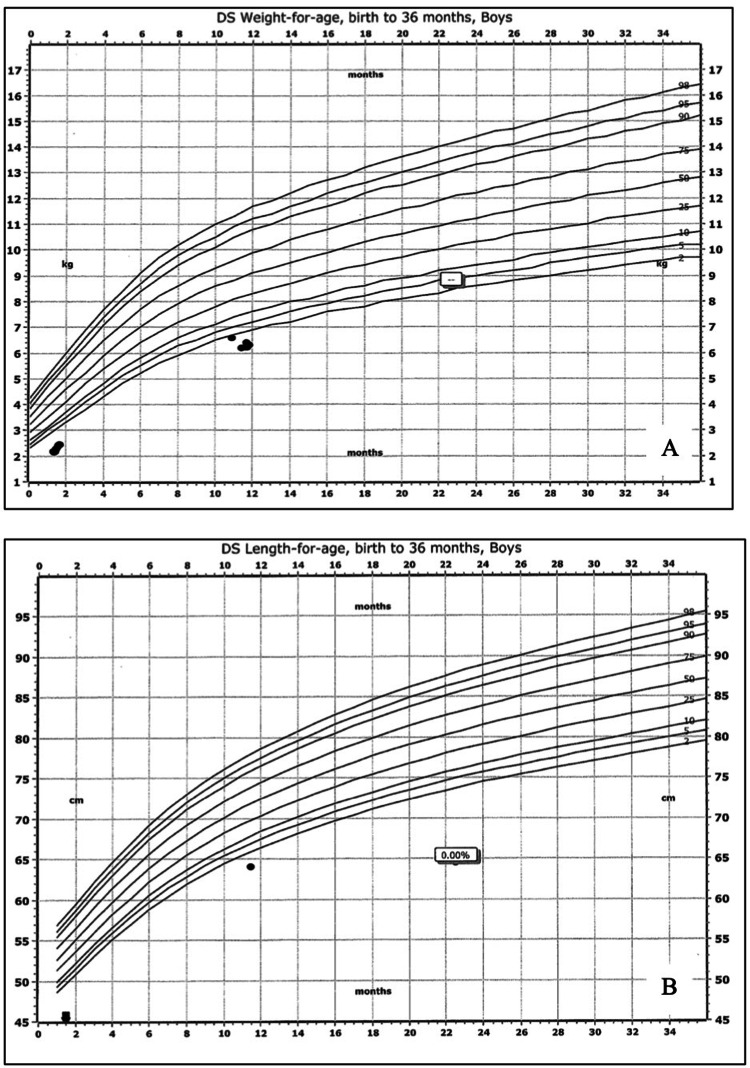
(A) and (B) represent the patient’s weight and length in the Down syndrome population, respectively. Both figures demonstrate the patient’s failure to thrive

An 11-month-old male infant presented to the hospital with intractable vomiting and decreased oral intake and urine output. His past medical history was notable for prematurity at 30 weeks’ gestation (twin pregnancy) and DS that had been diagnosed at four weeks of age. Further, the patient had a two-month history of intermittent spit-ups, which had prompted adjustments in his formula. Despite the feeding change, parents noted that his spit-ups had appeared to increase in frequency and intensity and become intractable after each feeding two days prior to the presentation. The patient also exhibited decreased oral intake and urine output. On presentation, he was afebrile with a rapid heart rate of 189 beats per minute; he had a respiratory rate of 24 breaths per minute with an oxygen saturation of 92% on room air. His growth chart was below the fifth percentile for weight and length on the DS scale (Figures 1A, 1B).

Physical exam was notable for fatigue, dry mucous membranes, and a delayed capillary refill. The characteristic facial dysmorphisms of DS were noted. Laboratory evaluation revealed a normal sodium of 146 mg/dL, potassium of 4.4 mg/dL, and chloride of 102 mg/dL. An acute kidney injury (AKI) was also diagnosed based on his blood urea nitrogen of 55 mg/dL and creatinine of 0.9 mg/dL. Additionally, his calcium was elevated at 14 mg/dL. After fluid resuscitation with one bolus of weight-based normal saline, his AKI resolved. However, his calcium remained abnormal at 12.9 mg/dL. In the setting of his hypercalcemia, the patient was admitted for further management.

**Figure 2:**
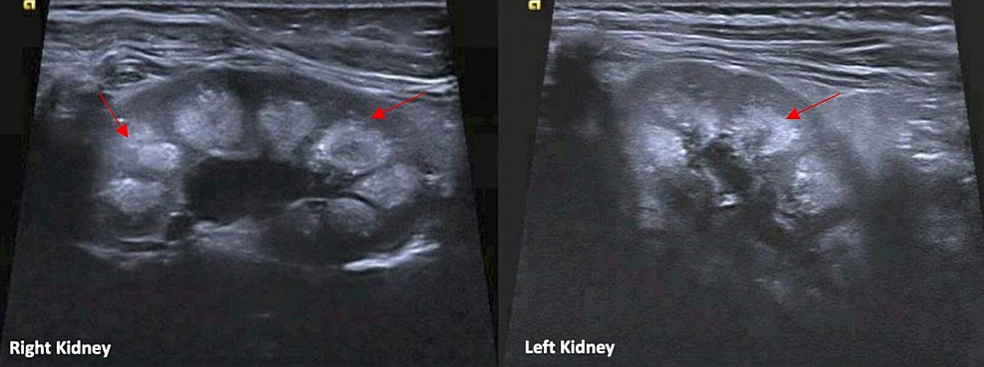
Both images depict the renal ultrasound showing moderate bilateral nephrocalcinosis (red arrows)

In the presence of hypercalcemia, the patient's parathyroid hormone (PTH) was appropriately low at 5.7 pg/mol (reference range: 12-88 pg/mol). His thyroid-stimulating hormone, free T4, 1,25-dihydroxyvitamin D, and 25-hydroxyvitamin D were all normal. His alkaline phosphatase (ALP) was low at 73 U/L. This prompted a workup for hypophosphatasia, a rare inherited disorder impacting bone and teeth mineralization through a loss-of-function mutation in the ALPL gene [3]. This was ruled out based on normal vitamin B6, ALP isoenzymes, and minimally elevated phosphoethanolamine. His parathyroid hormone-related peptide (PTHrP) was high at 15.3 pmol/L (reference range: <2.5 pmol/L). This raised a concern for malignancy and led to an extensive evaluation. His lactate dehydrogenase, uric acid, human chorionic gonadotropin, homovanillic acid, and vanillylmandelic acid for neuroblastoma were all normal. A neck ultrasound (US) along with a CT of the chest, abdomen, and pelvis did not identify any suspicious mass. The skeletal survey demonstrated normal bone mineralization. Therefore, the high level of PTHrP was concluded to be related to his AKI, rather than an underlying cancerous process. Urine studies demonstrated an elevated urinary excretion of calcium and a follow-up renal US showed bilateral medullary nephrocalcinosis (Figure 2).

As a result, he was suspected to have hypercalcemia in infants with Down syndrome, also known as ABCD syndrome.

He was briefly started on calcitonin to stabilize his elevated calcium level. Once the diagnosis of ABCD syndrome was made, he began a low-calcium and vitamin D-free formula. Meanwhile, he was on potassium phosphate and vitamin D supplementation. He also received Bicitra (sodium citrate/citric acid) to address his nephrocalcinosis. During his follow-up visit with a pediatric endocrinologist, his serum calcium, potassium, and phosphorus were found to have normalized. However, the patient struggled to gain weight and further evaluation with a registered dietitian was advised.

## Discussion

ABCD syndrome is a rare condition, with only six case reports published in the literature so far [4-9]. It is now regarded as a cause of hypercalcemia in children less than two years of age [1]. Table 1 summarizes the details of the DS patients who have been featured in the seven case reports, including our case, published so far on ABCD syndrome.

**Table 1:** Demographic and clinical features in reported cases of hypercalcemia in Down syndrome patients Ca: calcium; Cr: creatinine; F: female; FTT: failure to thrive; M: male; mo: months old; S/S: signs and symptoms

Case	Age, sex	Presenting S/S	Creatinine (ref range: 0.2-0.7 mg/dL)	Peak calcium (ref range: 8.6-10.3 mg/dL)	Urinary Ca/Cr ratio (ref range: <0.14)	Medullary nephrocalcinosis
Filler et al. [8]	10 mo, F	Vomiting and FTT	0.689	Ionized Ca of 7.17 (ref range: 3-4.4 mg/dL)	2.05	Yes
Cobeñas et al. [6]	15 mo, F	FTT	0.4	12	1.84	Yes
Andreoli et al. [5]	18 mo, F	FTT	1	14.4	1.11	Yes
Tran et al. [4]	24 mo, M	FTT	0.5	12	1.74	Yes
Ramage et al. [7]	33 mo, F	Incidental	1.13	13	2.24	Yes
Proesmans et al. [9]	48 mo, F	FTT	1.9	13.4	2.03	Yes
Our case	11 mo, M	Vomiting and FTT	0.9	14	0.83	Yes

Failure to thrive is the most common presenting symptom of the condition, observed in six out of seven patients. Vomiting is another frequently seen symptom. Renal impairment is also prevalent in this condition, likely due to a combination of high calcium load and nephrocalcinosis. Given the rarity of this disorder, a diagnostic guideline is not yet established. As the name implies, the diagnosis of this syndrome relies on detecting hypercalcemia, hypercalciuria, and nephrocalcinosis in DS patients. A renal US is useful in detecting nephrocalcinosis, which was documented in all cases of ABCD syndrome reported so far. It is important to point out that the constellation of hypercalcemia, hypercalciuria, and nephrocalcinosis can be seen in other disorders such as Williams syndrome, thyrotoxicosis, hyperparathyroidism, sarcoidosis, and certain malignancies as well [7]. Therefore, providers must consider and rule out other causes before establishing a diagnosis. Currently, the pathophysiology of ABCD syndrome is not fully understood. Suggested underlying mechanisms include excessive calcium intake, a genetic predisposition to augment calcium absorption, a renal tubular disorder, and increased sensitivity to 25-hydroxyvitamin D [4,5,7,10]. Dietary restriction of calcium is the treatment of choice. ﻿Biochemical and clinical improvement following dietary calcium restriction has been noted in all patients. In fact, a positive response to calcium restriction has been recommended to be a major criterion for the diagnosis of ABCD syndrome [4].

## Conclusions

ABCD syndrome is a very rare condition, and only six case reports on the condition have been published in the literature so far. Typically, DS patients present with failure to thrive and/or vomiting. Evaluation usually reveals hypercalcemia, hypercalciuria, and nephrocalcinosis on the renal US. AKI can often be seen. ABCD is a diagnosis of exclusion. Therefore, it is vital to rule out other causes of hypercalcemia before establishing a diagnosis. Dietary restriction of calcium is the treatment of choice, which can result in the normalization of serum calcium levels.
